# Expression of concern: Decoration of graphene oxide nanosheets with carboxymethylcellulose hydrogel, silk fibroin and magnetic nanoparticles for biomedical and hyperthermia applications

**DOI:** 10.1039/d4na90100b

**Published:** 2024-09-18

**Authors:** Mostafa Ghafori Gorab, Hooman Aghamirza Moghim Aliabadi, Amir Kashtiaray, Mohammad Mahdavi, Milad Salimi Bani, Andisheh Etminan, Nabi Salehpour, Reza Eivazzadeh-Keihan, Ali Maleki

**Affiliations:** a Catalysts and Organic Synthesis Research Laboratory, Department of Chemistry, Iran University of Science and Technology Tehran 16846-13114 Iran maleki@iust.ac.ir reza.tab_chemist@yahoo.com +98-21-73021584 +98-21-73228313; b Advanced Chemical Studies Lab, Department of Chemistry, K. N. Toosi University of Technology Tehran Iran; c Endocrinology and Metabolism Research Center, Endocrinology and Metabolism Clinical Sciences Institute, Tehran University of Medical Sciences Tehran Iran; d Department of Biomedical Engineering, Faculty of Engineering, University of Isfahan Isfahan Iran; e School of Mechanical Engineering, Iran University of Science and Technology (IUST) Tehran Iran; f Department of Medical Biotechnology, Pasteur Institute of Iran Tehran Iran

## Abstract

Expression of concern for ‘Decoration of graphene oxide nanosheets with carboxymethylcellulose hydrogel, silk fibroin and magnetic nanoparticles for biomedical and hyperthermia applications’ by Mostafa Ghafori Gorab *et al.*, *Nanoscale Adv.*, 2023, **5**, 153–159, https://doi.org/10.1039/D2NA00394E.

*Nanoscale Advances* is publishing this expression of concern in order to alert our readers to the fact that concerns have been raised regarding the reliability of the hemolysis plate images in Fig. 5 and the MTT plate image in Fig. 6. An investigation is underway, and an expression of concern will continue to be associated with the article until a final outcome is reached.

Jeremy Allen

13th September 2024

Executive Editor, *Nanoscale Advances*

